# Effects of Acupuncture Applied to Sanyinjiao with Different Stimuli on Uterine Contraction and Microcirculation in Rats with Dysmenorrhea of Cold Coagulation Syndrome

**DOI:** 10.1155/2014/328657

**Published:** 2014-04-09

**Authors:** Wing-sze Hsu, Xiao-yu Shen, Jia-min Yang, Li Luo, Ling Zhang, Dan-dan Qi, Song-xi Shen, Shi-peng Zhu, Ya-fang Zhao, Xiao-xuan Ren, Meng-wei Guo, Xiao-hong Li, Bo Ji, Lu-fen Zhang, Jiang Zhu

**Affiliations:** School of Acupuncture and Moxibustion, Beijing University of Chinese Medicine, No. 11 Bei San Huan Dong Lu, Chao Yang District, Beijing 100029, China

## Abstract

In this study, we try to evaluate the effects of acupuncture stimulation with different amounts at Sanyinjiao (SP6) on uterine contraction and uterus microcirculation in rats with dysmenorrhea of cold coagulation syndrome and to explore whether there is direct relativity between “De qi” and needle stimulus intensity. Diestrus female rats were randomly divided into 4 groups, including saline control group, model control group, “A” stimulus group (with strong stimulus), and “B” stimulus group (with weak stimulus). We found that dysmenorrhea rats of the cold coagulation syndrome present a high intensity in uterine tension and high contraction of microvascular diameter. Acupuncture applied with two different stimuli could relieve the symptoms, but, compared with “B” stimulus, “A” stimulus leads to better outcomes on reducing uterine contraction and increasing diameter of uterine microvascular; moreover, hand manipulation during needling mediates the curative effect on the microvascular diameter. Our finding indicates that using thick needles and deep insertion with hand manipulation are more effective and achieve desired level of “De qi” in dysmenorrhea rats.

## 1. Introduction

“De qi” is a main concept in traditional Chinese acupuncture theory [1, 2]. It has been noticed by people since ancient times. In the book* Miraculous Pivot-Nine Types of Needle and Twelve Yuan-Primary Points*, the relationship between “De qi” and curative effect is mentioned that the key point of acupuncture therapy is to wait for the “qi” arrival, and to use manipulations to induce the needle sensation if the “qi” has not arrived. If the “qi” has arrived, stop stimuli and withdraw the needle [3]. Previous researches also proved the relationship between the acupuncture “De qi” and clinical efficacy [2, 4]. Concerning the evaluation of “De qi,” clinical research concludes that “De qi” can be felt by the operator himself and the patient with the needling sensation. When “qi” arrives the operator feels fish bite in fishing under the needle or needle fibrillation, and at the same time the patient has an aching numb or tingling sensation, electric shock, and so forth. In a word it is a feeling of both the operator and patient. Some patients preferred to request for stronger feeling of “De qi” sensation during the treatment [5].

On the other hand, some studies have found that the intensity of “De qi” is not only affected by the body state, the specificity of acupoints, and the intervention methods, but also closely related to the intensity of stimuli and the needle thickness, the depth of needling, and different manipulating methods [6–8]. Recently, more and more clinical and experiment researches show that the strength of needling sensation and the curative effect of some diseases are not positively correlated [9, 10]. This argument is not only emphasized by the new methods of needling, but also actually recorded in the ancient literature that the needling sensation is influenced by gender, age, nature of disease, specificity of acupoints, and so forth. Therefore, only pursuing the needling sensation in the treatment process and believing that the needling sensation is equal to “De qi” is lack of scientific basis. The book* Miraculous Pivot-Ni Shun Fei Shou* has mentioned, “Because young children have weak physique and insufficient “qi” and blood, it is suggested to use thin needles, shallow needling without needle retention”, “Skinny patients are easy to suffer from deficiency of “qi” and blood. They are suggested to use shallow needling with no retention of needles [11].” Therefore, in light of different physique states, shallow needling is suggested. Previous studies showed that the needling sensation in different diseases was not the same. In general, the “bi” syndrome, paralysis, hemiplegia and acute pain are suitable to use strong intensity of acupuncture, and for some diseases, such as insomnia and hemifacial spasm, mild needling sensation is enough. So in the treatment of spastic disorders the needle-embedding therapy is adopted with having slight stimuli only [12]. Clinical tests reveal that the effect of deep needling (depth: 10 mm) was better than shallow needling (depth: 3 mm) in relieving muscle pain [13]. Therefore, the depth of needling and the intensity of stimuli are considered according to gender, age, nature of disease, and specificity of acupoints. It clearly indicates that different needling sensation plays an important role in acupuncture “De qi” effect, and, especially in pain related diseases, we believed that strong stimulation can cause stronger “De qi” sensation and better treatment effect to relieving the pain.

In this experiment, we will use the dysmenorrhea of the cold coagulation syndrome rats and two different intensities of stimuli (“A” stimulus group with strong stimulus and “B” stimulus group with weak stimulus) to evaluate the relationship between the stimulation intensity (an indirect indicator of “De qi” sensation) and the treatment effect. We believe that the result obtained will enhance our understanding of the association among “De qi,” uterine contraction, and microcirculation changes.

## 2. Material and Methods

### 2.1. Experimental Animal

This experimental scheme was approved by the Beijing University of Chinese Medicine Ethics Committee on April 13, 2012 (approval number: 2012-040). The animal experiment of laboratory animal care was complied with the United States National Institutes of Health Advocacy, using the guidelines of reduction, replacement, and refinement of the animal experiment “3R” principle: adoption of clean grade Sprague-Dawley rats (SD), female, rats aged 3 months, weighing (240 ± 20) g, and sexual maturity but without mating; rearing conditions: indoor temperature (23 ± 1)°C, humidity (45 ± 5)%, general feed, and free drinking water.

### 2.2. Experimental Equipment and Reagents

The equipment and reagents used were estradiol benzoate injection, produced by Ningbo Sansheng Pharmaceutical Co. Ltd., batch number: 120207. oxytocin injection, produced by Shanghai Hefeng Factory, batch number: 120302, self-control of methylene blue staining solution, 20% Urad injection: batch number T20080530, BC/BD-379HB horizontal freezer (Qingdao Haier Group), BL-420F Biological Function Experimental System, Chengdu Taimeng Technology Co. Ltd, FT-100 Type Tension Transducer, Chengdu Taimeng Technology Co. Ltd, XW-B-3 Cold Light Microcirculation Tester, Nanjing Sheng Photoelectric Medical Instrument Co., Ltd, KEL-2000 Type Microscope Temperature Controller, Nanjing Sheng Photoelectric Medical Instrument Co., Ltd, and SG-H1 Type Constant Temperature and Humidity Test System, Nanjing Sheng Photoelectric Medical Instrument Co., ltd.

### 2.3. Experimental Groups

After screening the estrous cycle through vaginal smear test [14], 64 female Sprague-Dawley rats were randomly divided into two groups. The first group of 32 rats was used for uterine contraction experiment and the second group of 32 rats was used for uterine microcirculation experiment. Each group was divided into four smaller groups: the saline control group, dysmenorrhea with cold coagulation syndrome model group (model control group), “A” stimulus group, and “B” stimulus group, and each group had 8 rats.

### 2.4. Preparation of Dysmenorrhea Model with the Cold Coagulation Syndrome

The cold coagulation syndrome model is improved from Xu Shuyun's pharmacological experiment [15, 16]. Firstly, it should determine the estrous cycle stage of rats through the vaginal smear test; then dysmenorrhea rats were produced from the diestrus rats. The model control group, “A” stimulus group, and “B” stimulus group were all designed to use the general freezing method combined with the estradiol benzoate injection molding. All of the groups were given daily subcutaneous injection of estradiol benzoate for 10 consecutive days. On the first day and the 10th day they were injected 0.5 mg/day, and from the 2nd–9th day they were injected 0.2 mg/day. 1 hour after the last estradiol benzoate injection, additional intraperitoneal injection of oxytocin 2 U/rat was given. From the 1st–5th day, after injection of estradiol benzoate, the rats were put into a −25°C freezer and frozen for 4 hours/day except that there was a 5-second ventilation at the 2nd hour. The saline control group was given daily injection of the same dose of saline. Evaluation index of the model control group was done by distinguished behavior, such as chills, scrunch, hazy sleepy, dull eyes, and dull response, and writhing response was based on Schmauss's and Yaksh [17] standard. The behavioral score standard and uterine microcirculation diameter shrinkage was adopted as the successful evaluation index of the model preparation.

### 2.5. Acupoints

On the basis of the morphological, anatomical, and physiological characteristics of the rat model and referring to Lin Wen-zhu's experimental acupuncture [18], standard positioning acupoint, Sanyinjiao (SP6), was located 10 mm above the hindlimb medial malleolus front of the tibia and fibula.

### 2.6. Treatment Method

For “A” stimulus group with a thick needle (Φ 0.25 × 40 mm), acupuncture was applied to the rat's Sanyinjiao (SP6) in the depth of 4-5 mm with twirling for 30 seconds at the beginning and 10 minutes after insertion of the needle. For “B” stimulus group with a thin needle (Φ 0.18 × 13 mm), acupuncture was applied to the rat's SP6 in the depth of 1-2 mm but did not use any hand manipulation. After observing the effectiveness index of the rat's uterine contraction and microcirculation for 20 min, the needle was withdrawn.

### 2.7. Experimental Method and Process

#### 2.7.1. Uterine Contraction Test

On the 10th day after the injection of estradiol benzoate, the rats were given 20% urethane (0.7 mL/100 g) intramuscular injection for anesthesia; then an incision of 2-3 cm length was made, 0.5 cm apart from the abdominal midline. After pulling out the uterine, a thread line was penetrated 1 cm above the uterine horn bifurcation. The free-end of the line was attached to the tension sensor and BL-420F Biological Function Experimental System Recorder was used to record the uterine contraction wave. The model control group and the treatment group were directly infused with 2U oxytocin, and the saline group was infused with 2U normal saline. Results were recorded 20 min after acupuncture intervention for the treatment group.

#### 2.7.2. Uterine Microcirculation Test

The rats were given intramuscular injection of 20% urethane (0.7 mL/100 g) for anesthesia. After they were completely anesthetized, incision of 2-3 cm length was made, 0.5 cm apart from the abdominal midline, and one side of the uterine and its ligament were pulled out. The model group and the treatment group had uterine infusion, 2U oxytocin, and the saline group had uterine infusion, 2U normal saline. Acupuncture was applied to the treatment group. After fixing the uterine and ligament to a constant temperature and humidity box, XW-B-3 Cold Light Microcirculation tester was used to observe the parameters every 5, 10, and 20 min values in fixed visual field.

### 2.8. Experimental Indexes

The experiment of uterine contraction observation index includes uterine contraction wave number, uterine contraction wave peak, and uterine activity (uterine contraction wave number × peak); uterus microcirculation, cap diameter, and blood flow status were observed under 40x magnification vision of the microvascular diameter. Flow status score was taken according to Tian's [19] standard. The semiquantitative determination of blood flow classification was divided into 4 levels: level 0, fast blood flow with smooth cords and none or microparticles; level I, comparatively fast flow with obvious grainy particles; level II, slow blood flow with sand-like or swinging stagnation; level III, blood flow completely stagnated or invisible blood flow.

### 2.9. Statistical Management

SAS 9.3 software was used for statistical analysis. Excel was used for graph generation. Data was represented into $\bar{X}\pm \text{SD}$. One-way ANOVA was used to analyze the difference between the two groups, and least-significant difference test was followed for the individual differences between the two groups; correlation analysis used the bivariate test and the range of correlation coefficient is −1 ≤ *R* ≤ 1, using *P* < 0.05 as the significant difference between the standard.

## 3. Results

### 3.1. Effects of Different Intensities of Acupuncture Stimuli on Rat's Uterine Contraction Degree in Each Period

**Figure 1:**
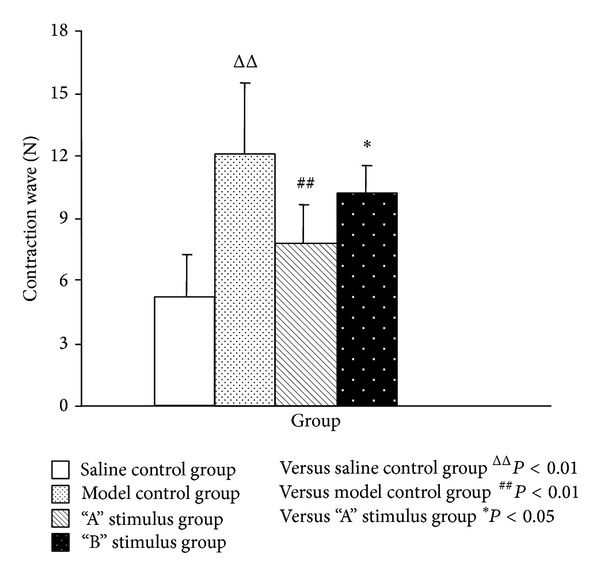
Effects of acupuncture with different stimuli on uterus contraction wave of female rats $\left(\bar{X}\pm \text{SD}\right)$. Compared with the model group, the number of contraction waves of “A” stimulus group was significantly decreased (*P* < 0.01) and “B” stimulus group also decreased but without statistical significance (*P* > 0.05).

**Figure 2:**
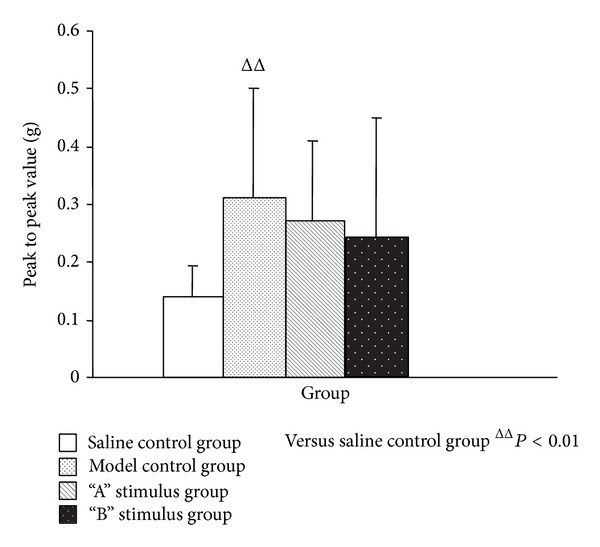
Effects of acupuncture with different stimuli on uterus contraction wave peak to peak value of female rats $\left(\bar{X}\pm \text{SD}\right)$. Compared with the saline control group, the peak value of model group was significantly increased (*P* < 0.01); although the result of both “A” stimulus group and “B” stimulus group compared with the model group has no statistical significance (*P* > 0.05), the trend was decreasing.

**Figure 3:**
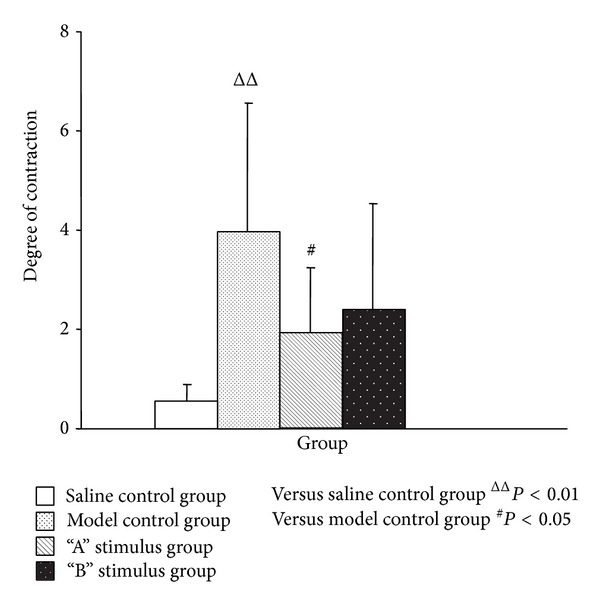
Effects of acupuncture with different stimuli on uterus degree of contraction of female rats $\left(\bar{X}\pm \text{SD}\right)$. Compared with the saline control group, the contraction degree of model group was significantly increased (*P* < 0.01) and compared with the model group the result of “A” stimulus group was decreased (*P* < 0.05); although “B” stimulus group has no statistical significance (*P* > 0.05), the trend was decreasing.

The number of uterine contraction wave, uterine contraction wave peak, and uterine activity of the model control group, compared with the saline control group, was significantly increased (*P* < 0.01) (Figures 1, 2, and 3). Compared with model control group, the uterine contraction wave number (*P* < 0.01) and uterine activity (*P* < 0.05) significantly decreased in “A” stimulus group. This result showed significant difference compared with “B” stimulus group (*P* < 0.05) in the decrease of uterine contraction wave number. Compared with the model control group, the uterine contraction wave number and uterine activity decreased in “B” stimulus group, but the difference had no statistical significance (Figures 1–3).

### 3.2. Effects of Different Intensities of Acupuncture Stimuli on the Rat's Uterine Microvascular Diameter in Each Period

**Figure 4:**
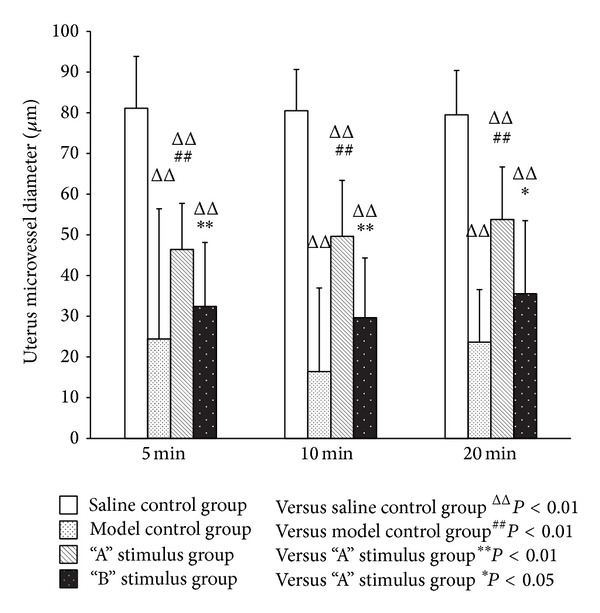
Effect of acupuncture with different stimuli on uterus mircovessel diameter of female rats at different periods $\left(\bar{X}\pm \text{SD}\right)$. Compared with the model group, “A” stimulus group had significant difference in uterine microvascular diameter expansion (*P* < 0.01). After acupuncture intervention, “B” stimulus group also had microvascular diameter expansion, but the difference was not statistically significant (*P* > 0.05). “A” stimulus group had significant difference compared with “B” stimulus group in every period, 5 min and 10 min (*P* < 0.01) and 20 min (*P* < 0.05).

**Figure 5:**
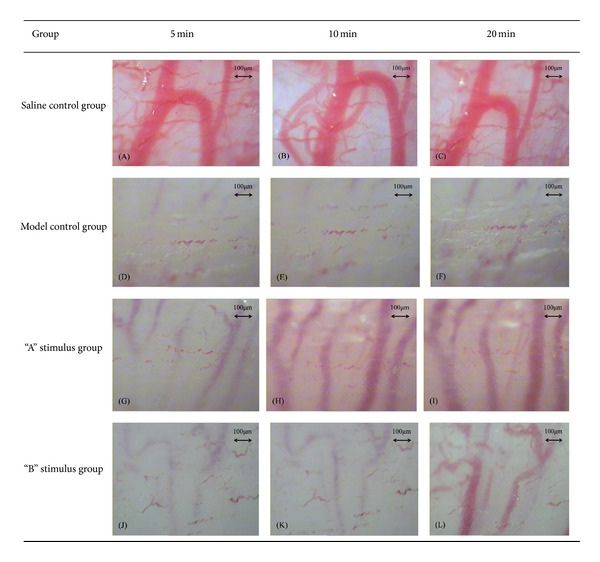
(A), (B), and (C) show the uterine microvascular diameter of saline control group under 40x magnification vision and the diameter nearly without any change during observation period. (D), (E), and (F) show the uterine microvascular diameter of model control group, the diameter nearly disappeared, and without any change during observation period. (G), (H), and (I) show the changes of uterine microvascular diameter of “A” stimulus group, especially after the hand manipulation of needle at 10 min as the diameter expanded significantly. (J), (K), and (L) show the changes of uterine microvascular diameter of “B” stimulus group, compared with the “A” stimulus group and “B” stimulus group without hand manipulation stimulus at 10 min, but at 20 min; we also can see that the diameter had expanded.

In every period, compared with the saline control group, the model control group and the treatment group had significant difference in uterine microvascular diameter contraction (*P* < 0.01). Compared with the model group, “A” stimulus group had significant difference in uterine microvascular diameter expansion (*P* < 0.01). After acupuncture intervention, “B” stimulus group also had microvascular diameter expansion, but the difference was not statistically significant (*P* > 0.05). “A” stimulus group had significant difference compared with “B” stimulus group in every period, 5 min and 10 min (*P* < 0.01) and 20 min (*P* < 0.05) (Figures 4 and 5).

### 3.3. Effects of Different Intensities of Acupuncture Stimuli on the Rats' Uterine Capillary Diameter in Each Period

**Figure 6:**
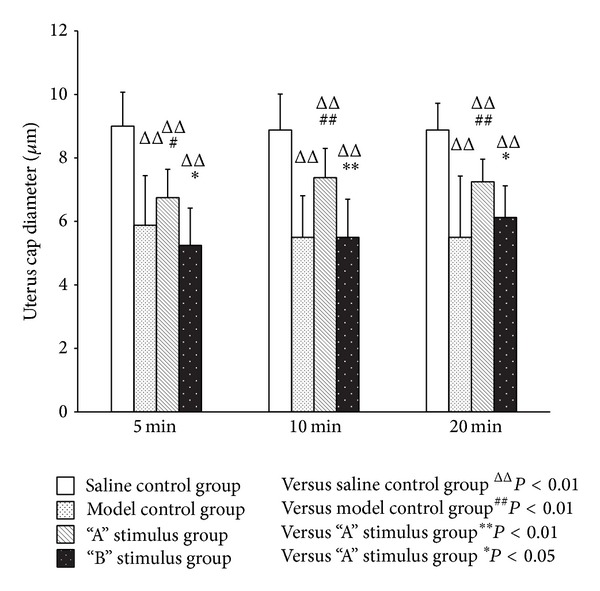
Effect of acupuncture with different stimuli on uterus cap diameter of female Rats at different periods $\left(\bar{X}\pm \text{SD}\right)$. Compared with the model group models, “A” stimulus group had significant expansion in uterus diameter of capillaries immediately after acupuncture treatment, 5 min (*P* < 0.05) and 10 min and 20 min (*P* < 0.01). After acupuncture stimulus, the expansion in uterine diameter of capillaries of “A” stimulus group, compared with “B” stimulus group, had significant difference at 5 min and 20 min (*P* < 0.05) and 10 min (*P* < 0.01). Compared with model control group, although “B” stimulus group had expansion too, the difference was not statistically significant (*P* > 0.05).

Uterine capillary diameter contraction of the model control group, compared with the saline control group, had significant difference (*P* < 0.01) in every period. Rat models in “A” stimulus group, compared with the model group, had significant expansion in uterus diameter of capillaries immediately after acupuncture treatment, 5 min (*P* < 0.05) and 10 min and 20 min (*P* < 0.01). Although “B” stimulus group had expansion too, the difference was not statistically significant (*P* > 0.05). After acupuncture stimulus, the expansion in uterine diameter of capillaries of “A” stimulus group, compared with “B” stimulus group, had significant difference: 5 min and 20 min (*P* < 0.05) and 10 min (*P* < 0.01). This indicated the effect of hand manipulation of needle in “A” stimulus group (Figure 6).

### 3.4. Effects of Different Intensities of Acupuncture Stimuli on the Rat's Uterine Microvascular Blood Flow in Each Period

**Figure 7:**
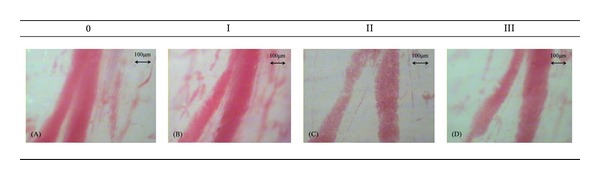
Blood flow classification. (A) Level 0, blood flow is fast, smooth cords, no or microparticles; (B) level I, flow faster, has obvious grainy; (C) level II, blood flow is slow, a sand-like, flow slowly; (D) level III, stagnant blood flow or not visible.

Compared with the saline control group, the model control group increased significantly in microvascular blood flow velocity (*P* < 0.01); compared with the model control group, “A” stimulus group improved significantly in uterine microvascular blood flow after acupuncture stimuli (*P* < 0.01). In “B” stimulus group, although the blood flow velocity increased, the difference was not statistically significant (*P* > 0.05). After acupuncture treatment, the microvascular blood flow of “A” stimulus group was significantly faster than “B” stimulus group, (*P* < 0.01) (Table 1, Figure 7).

Table 1 shows the effect of acupuncture with two different stimuli on uterus blood flow of female rats at different period. Compared with the model control group, “A” stimulus group improved significantly in uterine microvascular blood flow after acupuncture stimuli (*P* < 0.01). In “B” stimulus group, although the blood flow velocity increased, the difference was not statistically significant (*P* > 0.05).

### 3.5. Analysis of the Correlation between the Uterine Contraction and Uterine Microcirculation

The relationship of uterine microvessel capillary diameter and the contraction of uterus showed negative correlation (*r* < −1). This proposes that, under dysmenorrhea condition, contraction of uterus and microcirculation are directly connected (Table 2).

Table 2 shows the relationship of uterine microvessel capillary diameter and the contraction of uterus had negative correlation (*r* < −1).

## 4. Discussion

### 4.1. Relationship between Uterine Tension and Uterus Microcirculation under Dysmenorrhea

Dysmenorrhea refers to painful menstruation, that is, recurrent abdominal pain or pain in the lumbosacral region in periods, before or after periods. According to the theory of Chinese medicine, it is induced by either “qi” stagnation and blood stasis, or cold coagulation and blood stasis, belonging to the heat accumulation type, liver and kidney injury type, and “qi” and blood deficiency type. The syndrome of cold coagulation and blood stasis is mostly seen [20]. Intense of uterine tension is closely related to the activity of the uterine smooth muscles [21]. In normal menstrual period, the basic tension of the uterine cavity is less than 1.33 kPa, the uterine pressure is less than 16 kPa, and the contraction frequency is 3~4 times every 10 min. However, during dysmenorrhea, the basic tension of the uterine cavity increases, the uterine contraction pressure goes up to 16~20 kPa, and the frequency is higher than 5 times every 10 min. Moreover, the rhythm of shrinkage is discorded. After the contraction, it is hard to relax completely [22]. The intense of abnormal uterine contraction could induce microcirculation disorder with uterine ischemia and hypoxia, which would cause dysmenorrhea [23].

During the menstrual period, because of the lysosomal instability, it is easy to cause endometrial cell lysis, resulting in endometrial tissue necrosis and release of large amounts of PGE_2_ and PGF_2*α*_; PGF_2*α*_ releases more than PGE_2_ which combined with PGF_2*α*_ receptor in the uterine spiral artery wall and executes the contraction of uterine smooth muscles. According to researches [24], the content of PG in endometrium of dysmenorrheal patients is higher than healthy women, and PGF_2*α*_ : PGE_2_ ratio is significantly greater as well. This results in uterine local vasoconstriction, blood flow decrease, blood supply deficiency, muscle ischemia, metabolite accumulation, pain, and uterine pressure exceeding the average arterial blood pressure. Consequently, the uterine cavity pressure increases and the uterus wall blood flow reduces, so the microcirculation gets disordered and finally causes dysmenorrhea [25].

According to the data, this study has found that dysmenorrhea of the cold coagulation syndrome presents a high intensity of uterine tension and high contraction of the microvascular diameter, the uterine microcirculation, and tension have negative correlation (*r* < −1). The uterine microvascular and capillary contraction decreases the uterine microvascular blood flow and increases uterine contraction wave number. This furthermore explains that dysmenorrhea could cause uterine muscle spasm, increase contraction, and produce microcirculation disorder. Accordingly, it proves that uterine contraction and uterus microcirculation are closely related to dysmenorrhea environment.

### 4.2. Different Acupuncture Stimuli Intensity Researches

Human body is a complex medium and acupuncture functions in a dynamic process. Acupuncture induces stress and strain on the soft tissues and creates “De qi.” When the inserted needle is twirled, the stimulus feeling of the connective tissue gets heavier. It is because the connective tissue contains a large number of collagen fibers, when the needle twisting, the collagen fibers winding on the needle and towing the nerve of blood vessels, patient will feel an aching numb or tingling sensation, electric shock, and so forth, which we know as “De qi” sensation [26]. Acupuncture treatment pursues the needling sensation. However, as the object of this experiment is dysmenorrhea rats of the cold coagulation syndrome, they cannot subjectively tell us their “De qi” feeling. Therefore we divided the effect factors into three points: (1) the depth of injection, (2) the thickness of needle, (3) and the hand manipulation, which can objectively evaluate the relationship between the “De qi” effectiveness and the treatment effect. There are studies about the different depths of acupuncture needling at Zusanli (ST36) on healthy rats. They have found that the index of thymus and the lymphocyte proliferation of the muscle group (needle piercing into ST36 muscle layer) is significantly higher than the subcutaneous group (needling obliquely thrusting into ST36 at 15° angle) and the normal group. This informs us that the needle inserted into ST36 must reach a certain depth (muscle layer) to increase the level of immunity. This shows that needling with different depths has direct impact on the treatment effect [27]. Moreover, there are studies using strong stimuli (vibrating frequency 200/min, each time 3 min for 3 sessions of treatment) applied to Neiguan (PC6) in the treatment of myocardial ischemia reperfusion injury (MIRI) rat models. As the result, concerning the curative effect on serum IMA and 5-HT spinal cord of the hypothalamus, the strong stimulus group is better than the mild stimulus group (without any stimulation) [28].

Based on this experimental study, the “A” stimulus group has better effectiveness than the “B” stimulus group in relieving dysmenorrhea of the cold coagulation syndrome in rats, such as uterine microcirculation diameter, blood flow, and the contraction of uterus and uterine activity. The “A” stimulus group receiving acupuncture for 5 minutes immediately presents some effects on tiny blood vessels, capillaries diastolic, and blood flow, which is greater than “B” stimulus group. After 10 minutes, the expansion of diameter of “A” stimulus group is better than “B” stimulus group, and has significant statistic difference. This further explains that hand manipulation can help the microvessel to relax. Besides, the “A” stimulus group can noticeably reduce uterine contraction and activity (*P* < 0.01), which means that using thick needles, deep insertion, and hand manipulation can alleviate spasmodic contraction of uterine muscles and can easily achieve “De qi.” In addition, we have found that, although the therapeutic effect in “B” stimulus group compared to the “A” stimulus group is not significantly different, there still some therapeutic effects, which explains the reason why some superficial needling, such as abdominal needle therapy, is also effective [29].

## 5. Conclusion

Dysmenorrhea of the cold coagulation syndrome presents a high intensity of uterine tension and high contraction of the microvascular diameter. Acupuncture applied with two different stimuli could relieve the symptoms, but, compared with “B” stimulus, “A” stimulus leads to better outcomes on reducing uterine contraction and increasing diameter of uterine microvascular. Moreover, we have found that hand manipulation can help to expand the microvascular diameter, which implies that hand manipulation is one of the important factors affecting the curative effect. Our finding highlights using thick needles and deep insertion with hand manipulation to cause appropriate intensity of stimuli to achieve “De qi” state in acupuncture treatment of dysmenorrhea. Results of this experiment remind us that in the treatment of dysmenorrhea with acupuncture appropriate but comparatively strong stimuli with particular hand manipulation can have better therapeutic effect.

## Figures and Tables

**Table 1:** The effect on uterus blood flow of female rats with different acupuncture stimuli at different periods.

Group	*N*	5 min					10 min					20 min				
		0	I	II	III		0	I	II	III		0	I	II	III	
Saline control group	8	8	0	0	0		8	0	0	0		8	0	0	0	
Model control group	8	0	0	2	6	ΔΔ	0	0	2	6	ΔΔ	0	0	2	6	ΔΔ
“A” stimulus group	8	0	3	5	0	ΔΔ##	0	3	5	0	ΔΔ##	0	1	7	0	ΔΔ##
“B” stimulus group	8	0	0	3	5	ΔΔ∗∗	0	0	3	5	ΔΔ∗∗	0	0	1	7	ΔΔ∗∗

Note. versus saline control group, ^ΔΔ^ *P* < 0.01; versus model control group, ^##^ *P* < 0.01; versus “A” stimulus group, **P < 0.01, and *P < 0.05.

**Table 2:** Correlation analysis of uterine contraction and uterine microcirculation.

*R*	Contraction wave	Peak to peak value	Degree of contraction
Uterus microvessel diameter 5 min	−0.60^ΔΔ^	−0.33	−0.47^ΔΔ^
Uterus microvessel diameter 10 min	−0.63^ΔΔ^	−0.35	−0.48^ΔΔ^
Uterus microvessel diameter 20 min	−0.62^ΔΔ^	−0.43	−0.56^ΔΔ^
Uterus cap diameter 5 min	−0.62^ΔΔ^	−0.17	−0.34^Δ^
Uterus cap diameter 10 min	−0.58^ΔΔ^	−0.22	−0.41^ΔΔ^
Uterus cap diameter 20 min	−0.59^ΔΔ^	−0.11	−0.27

Note. ^ΔΔ^ *P* < 0.01,^Δ^ *P* < 0.05.
